# Intradural *Candida Albicans* infection that presented as epidural abscess: A case report^☆^

**DOI:** 10.1016/j.ijscr.2023.108337

**Published:** 2023-05-18

**Authors:** Christopher Lucasti, Maxwell M. Scott, Emily K. Vallee, Joseph Kowalski

**Affiliations:** a UBMD Orthopaedics and Sports Medicine Doctors Buffalo, Buffalo, NY, United States of America; b Jacobs School of Medicine and Biomedical Sciences Buffalo, NY, United States of America

**Keywords:** Intradural infection, Epidural presentation, Case report

## Abstract

**Introduction:**

Intradural *Candida Albicans* infections are rare with limited number of reports on the pathological condition. Among these reports, patients with these infections had radiographic evidence supporting an intradural infection diagnosis. In this case, the patient displayed radiographic imaging suggestive of an epidural infection, but surgery revealed the infection to be intradural. This case exemplifies the importance of considering intradural infections in future cases of suspected epidural abscesses and highlights antibiotic management of intradural *C. albicans* infections.

**Presentation of case:**

A incarcerated 26-year-old male presented with a rare *Candida Albicans* infection. He arrived at the hospital unable to walk, and radiographic imaging was consistent with a thoracic epidural abscess. Due to his severe neurologic deficit and spreading edema, surgical intervention was required and revealed no signs of epidural infection. Incision of the dura revealed a purulent material cultured as *C. albicans*. After six weeks, the intradural infection returned and the patient required another surgery. This operation helped prevent further motor function loss.

**Discussion:**

When patients present with a progressive neurologic deficit and radiographic evidence indicative of an epidural abscess, it is important for surgeons to be mindful of a possible intradural infection. If no abscess is found in the epidural space during surgery, opening the dura in patients with worsening neurologic symptoms must be considered to rule out an intradural infection.

**Conclusion:**

Preoperative suspicion of an epidural abscess can differ from intraoperative diagnosis and looking intradural for an infection can prevent further motor loss.

## Highlights

• A perplexing and rare *spinal C. albicans* infection posed a diagnostic challenge, as preoperative imaging differed from intraoperative findings.
• Surgeons must be proactive in spine cases to address potential rapid neurological decline. Dural incisions may be necessary in rare spinal *C. albicans* infection cases.
• Swift intervention with amphotericin B is crucial for spinal *C. albicans* infections, especially if fluconazole fails to yield significant improvement.

## 1 Introduction

Spinal infections by *Candida* species are rare. *Candida* is part of the endogenous flora that colonizes the mouth, bowel, vaginal mucosa, and skin surfaces. Of the *Candida* species, *Candida albicans* is the most common [1]. Superficial infections such as oral and vaginal yeast infections are among the most prevalent type, whereas immunocompromised individuals have a higher risk of developing severe and systemic complications [2]. These complications include fungal pneumonia, meningitis, endocarditis, endophthalmitis, osteoarticular infection, and urinary tract infections [3].

Spinal involvement is rare with limited reports in literature examining this type of infection. Vertebral osteomyelitis is thought to occur primarily through hematogenous dissemination of *Candida* due to the substantial vascular supply surrounding the vertebrae and discs [4]. This suspicion is supported by the fact that 60 % of *Candida*-related osteomyelitis occurs in the spine [5,6]. Patients with Candida-related vertebral osteomyelitis may present with multiple weeks of back pain, fever, or neurological deficits [4]. This manuscript was prepared with the SCARE 2020 guidelines [7].

## 2 Case

In May of 2013, a twenty-six-year-old male patient was involved in a motor vehicle accident. Cervical MRI at the time of the accident revealed multiple herniated discs. Following this accident, the patient was incarcerated. While in prison, he complained of back pain and was administered a corticosteroid injection. Soon after the injection, the patient reported increased bilateral lower extremity weakness. He denied any bladder or bowel control problems, fever, nausea, chills, or vomiting. In September of 2013, following two months of bilateral lower extremity weakness with difficulty ambulating, he was brought to the hospital for further evaluation.

The twenty-six-year-old patient had a history of cocaine addiction, used non-steroidal anti-inflammatory (NSAIDs) frequently for back pain, and denied using intravenous drugs. He may have received multiple intramuscular methylprednisone while in prison for his back pain. Additionally, the patient was known to be positive for hepatitis C, with an unknown etiology.

**Fig. 1:**
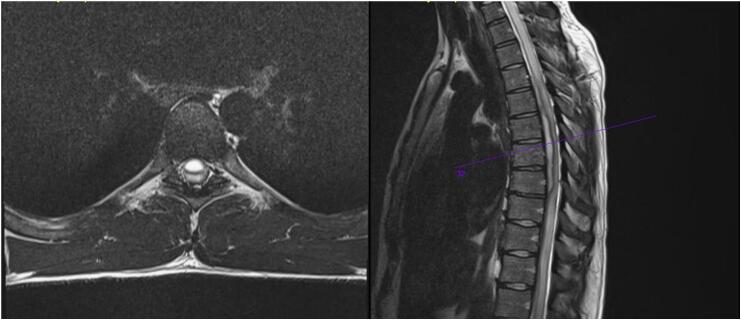
Axial/sagittal T2 MRI demonstrating ventral abscess. Mass appeared to be epidural and measured at least 6 mm × 10 cm in length. Gross distortion of the spinal cord with local edema as well as menial enhancement through the thoracic spine can be seen.

An MRI of the spine showed a sizeable epidural abscess at the T3-T7 level (Fig. 1). Besides elevated C-reactive proteins, his blood work was normal. The patient was started on Vancomycin and Metronidazole. However, due to the size of the abscess, edema beginning to spread to the cervical region, and neurologic deficits, surgical intervention was scheduled for the following day.

In the operating room, laminectomies were performed from T6 to T8. Upon exposure of the epidural space, no pus formation was found as demonstrated on MRI. The pedicle of T7 was removed to allow exploration of the ventral epidural space and the space contained no evidence of abscess, tumor, or phlegmon. Due to the patients declining motor function, a durotomy from T6-T8 was performed. Purulent material was seen upon incision and no spinal fluid was appreciated. Because of the severe edema, primary repair of the dura was not possible. Distally the dura was approximated, and a duroplasty was performed for closure of T6-T7.

Despite the operation, the patient's neurologic function did not improve as they still had no below sensation below the T5 dermatome and no lower extremity motor control. However, we believe our high suspicion of an intradural infection during operation prevented further morbidity and mortality as we were able to decompress the spinal cord and get a culture for proper treatment of the infection. Labs revealed the infecting organism was *C. albicans* and the patient began 800 mg of IV fluconazole daily. After a week of intravenous Fluconazole, the patient was transferred to the hospital's rehabilitation center and started on 800 mg oral doses of the medication. The patient was stable, and an MRI of the thoracic spine demonstrated improvement.

**Fig. 2:**
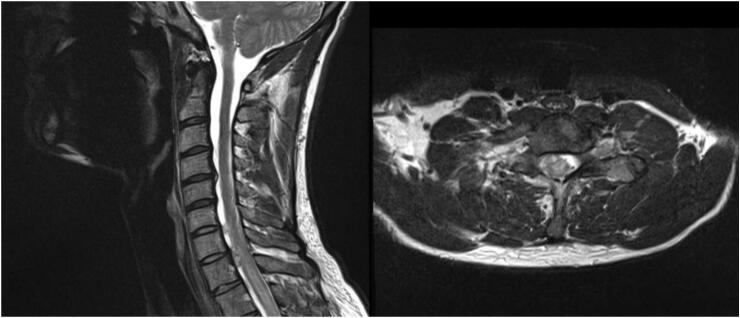
Axial/sagittal T2 weighted MRI revealing ventral compression at the C7-T1 disc space, along with retrovertebral compression.

Six weeks after the index surgery, the patient began having pain at the base of his neck and radiating down his arm. His blood work showed an elevated erythrocyte sedimentation rate and increased C-reactive protein levels. An MRI of the cervical spine revealed a mass at T1 that was not present in the MRI from October (Fig. 2).

The patient returned to the operating room for a T1 corpectomy to expose the dura, due to the fact the patient had retrovertebral compression. Visualization of the spinal cord revealed tense area with discoloration. A needle was placed into this portion of the dura, and aspiration revealed no fluid. The dura was therefore incised over this area and exposed a frank purulent material and granulation tissue. This material was cleared from the intradural space and sent for culture. The spinal cord was irrigated with standard saline solution before the dura was closed. The patient underwent a fusion between C7, T1, and T2. He was then transferred to the ICU in stable but critical condition. The cultures returned positive for *C. albicans*, which remained susceptible to fluconazole.

He maintained 5/5 motor strength in the upper extremities. To treat the *C. albicans* infection, the patient was started on 5 mg/kg per day of liposomal amphotericin B for six weeks. After this, he resumed 400 mg/day of fluconazole for two months.

The patient suffered from medical commodities in the following years, many related to his lower extremity paraplegia. These problems included multiple urinary tract infections, chronic kidney disease, and decubitus ulcers, some of which required surgical excisional debridement. The patient also lived with a catheter, a colostomy bag, and underwent a Girdlestone procedure for bacterial osteomyelitis of the hip. Additionally, a right iliacus abscess that tracked to the psoas was surgically evacuated. Ultimately, the patient became septic secondary to a presumed urinary tract infection and succumbed to his illness in 2019.

## 3 Discussion

Over the last few decades, fungal infections have seen an increase which poses patients at a greater risk for invasive spine infections. However, spine infections by *Candida albicans* still remain rare, and little literature has reported intradural infections with the species. When these infections do occur, they may present as a range of disorders, including osteomyelitis [1]. Patients with osteomyelitis of the spine usually present with non-specific symptoms including fever and back pain [6]. Ultimately, these symptoms can progress to neurological symptoms or deficits [4,6]. Neurologic deficits in infections have been contributed to multiple factors. First, disc narrowing can occur due to chronic inflammation. This condition, along with an epidural abscess, can lead to spinal cord compression and neurological symptoms [8]. Additionally, vasculitis due to infection has been described in the literature. This condition can lead to spinal cord ischemia and neurological deficits [9].

The significance of our patient lies in the fact that they serve as an illustration of how imaging and preoperative diagnosis can differ from intraoperative findings. His MRI and symptoms were indicative of an epidural abscess without meningeal involvement. He did not present with any fever, headache, nuchal rigidity, or altered mental status that would suggest meningitis [[10], [11], [12], [13], [14]]. In similar cases, radiologic findings illustrated intradural involvement [10,12,13]. In our case, surgery revealed an intradural infection.

Other surgeons have reported infections from different organisms where imaging reveals an epidural abscess, but the infection was found in the intradural space intraoperatively. One of these cases was from *Staphylococcus Aureus*. The patient presented with signs of meningeal involvement, but the MRI suggested an epidural abscess [15]. However, to our knowledge, this is the first reported case of *C. albicans* presenting as an epidural abscess, both clinically and radiographically, before being discovered intradural while in surgery.

Though the source of our patient's *C. albicans* infection was not determined, our case sparks discussion on ways spinal infections can occur. First, reports of steroid injections contaminated with fungal species can be found in the literature. In 2012, thousands of patients were potentially exposed to contaminated steroid injections. Most patients who presented with spinal infection, meningitis, and stroke had undergone epidural injection [16]. Second, forms of immunosuppression have been linked to invasive fungal infections [17]. Frazier et al. found over half of its patients in its study of fungal spine infections had some sort of immunodeficiency, including human immunodeficiency virus and corticosteroid use [18]. Lastly, intravenous drug use has been linked to intervertebral infection and osteomyelitis due to fungus [19].

In previous spinal *Candida* infection cases, patients underwent laminectomy in the affected area as defined by MRI and clinical signs. As with our patient's surgery, this exposure revealed no epidural mass and purulent material was discovered after dura incision [10,11,13]. In previous cases of intrathecal Candida infection, the purulent material within the dura was debrided and the cord was irrigated prior closing the dura.

Our case illustrates the importance of preparing for a possible intradural infection when treating a patient with a preoperative diagnosis of an epidural abscess. If no frank purulent material is seen upon exposure of the dura, we recommend dura incision in patients with progressive neurologic deficit. Although this surgical approach did not help the patient with their motor function after the first surgery, we believe the intradural presentation prior to the second operation allowed for faster surgical intervention and opening the dura to prevent further neurologic decline. In the other reported cases of intradural Candida infections early surgical intervention likewise resulted in a positive outcome [10,11,15].

Our case highlights the medical management for intradural *Candida* infections. After initial treatment with vancomycin and metronidazole, our patient was switched to IV fluconazole when intraoperative cultures were found to be susceptible to this anti-fungal agent. Khazim et al. described the success of surgical debridement and fluconazole for *C. albicans* osteomyelitis [6]. Despite the susceptibility to fluconazole, this treatment plan proved ineffective. Therefore, after the second surgery, liposomal amphotericin B was used for six weeks and then fluconazole for two months. Amphotericin B and flucytosine have demonstrated efficacy in the past for treating intradural spinal *C. albicans* infection [13]. Given the possibility for rapid deterioration, we recommend early consideration of amphotericin B if there is minimal improvement on fluconazole.

## 4 Conclusion

Our patient presented with a rare *C. albicans* infection that initially appeared epidural based on imaging and examination. However, during surgery, the abscess was found intrathecal. High clinical suspicion for an intradural infection can help prompt intervention and prevent further neurologic decline. If infection returns in the postoperative period, early surgical intervention can help prevent a further neurologic deficit. Additionally, we advocate for early consideration of amphotericin B if fluconazole appears ineffective, preventing the spread of the infection in patients at risk for severe and debilitating outcomes.

## Patient consent

Written informed consent was obtained from the patient for publication of this case report and accompanying images. A copy of written consent is available for review by the Editor-in-Chief of this journal on request.

## Ethical approval

This study is exempt from ethical approval at our institution (University at Buffalo) as patient consent was obtained.

## Funding

None.

## Author contribution

Christopher Lucasti- Study concept and design, writing of the paper

Maxwell Scott- Data collection, writing of the paper

Emily Vallee- Writing of the paper and review

Joseph Kowalski- Writing of the paper and review

## Guarantor

Christopher Lucasti

## Conflict of interest statement

None.

## References

[1] McLeod N., Fisher M., Lasala P.R. (2019). Vertebral osteomyelitis due to Candida species. Infection.

[2] Mayer F.L., Wilson D., Hube B. (2013). Candida albicans pathogenicity mechanisms. Virulence.

[3] Silva R.F. (2010). Chapter 8: fungal infections in immunocompromised patients. J. Bras. Pneumol..

[4] Miller D.J., Mejicano G.C. (2001). Vertebral osteomyelitis due to Candida species: case report and literature review. Clin. Infect. Dis..

[5] Gathe J.C. (1987). Candida osteomyelitis. Report of five cases and review of the literature. Am. J. Med..

[6] Khazim R.M., Debnath U.K., Fares Y. (2006). Candida albicans osteomyelitis of the spine: progressive clinical and radiological features and surgical management in three cases. Eur. Spine J..

[7] Agha R.A. (2020). The SCARE 2020 guideline: updating consensus surgical CAse REport (SCARE) guidelines. Int. J. Surg..

[8] Kulcheski A.L. (2015). Fungal spondylodiscitis due to Candida albicans: an atypical case and review of the literature. Rev. Bras. Ortop..

[9] Bhattacharyya S., Bradshaw M.J. (2021). Infections of the spine and spinal cord. Continuum (Minneap Minn).

[10] Argersinger D.P. (2019). Intradural cauda equina Candida abscess presenting with hydrocephalus: case report. J. Neurosurg. Spine.

[11] Sakayama K. (2002). Subdural spinal granuloma resulting from Candida albicans without immunosufficiency: case report. Spine (Phila Pa 1976).

[12] Lindner A. (1995). Magnetic resonance image findings of spinal intramedullary abscess caused by Candida albicans: case report. Neurosurgery.

[13] Roh J.E. (2011). Sequential magnetic resonance imaging finding of intramedullary spinal cord abscess including diffusion weighted image: a case report. Korean J. Radiol..

[14] Putz K., Hayani K., Zar F.A. (2013). Meningitis. Prim. Care.

[15] Cheon J.E. (2015). Pyogenic Intradural abscess of lumbar spine: a case report. Korean J. Neurotrauma.

[16] Kauffman C.A., Malani A.N. (2016). Fungal infections associated with contaminated steroid injections. Microbiol. Spectr..

[17] Mei-Sheng Riley M. (2021). Invasive fungal infections among immunocompromised patients in critical care settings: infection prevention risk mitigation. Crit. Care Nurs. Clin. North Am..

[18] Frazier D.D. (2001). Fungal infections of the spine. Report of eleven patients with long-term follow-up. J. Bone Joint Surg. Am..

[19] Rowe I.F. (1988). Intervertebral infection due to Candida albicans in an intravenous heroin abuser. Ann. Rheum. Dis..

